# Search for a Neutron Electric Dipole Moment

**DOI:** 10.6028/jres.110.019

**Published:** 2005-06-01

**Authors:** R. Golub, P. R. Huffman

**Affiliations:** Physics Department, North Carolina State University, Raleigh, NC 27695 USA

**Keywords:** electric dipole moment, superthermal production, ultracold neutron

## Abstract

The possible existence of a nonzero electric dipole moment (EDM) of the neutron is of great fundamental interest in itself and directly impacts our understanding of the nature of electro-weak and strong interactions. The experimental search for this moment has the potential to reveal new sources of T and CP violation and to challenge calculations that propose extensions to the Standard Model. The goal of the current experiment is to significantly improve the measurement sensitivity to the neutron EDM over what is reported in the literature. The experiment has the potential to either measure the magnitude of the neutron EDM or to lower the current experimental limit by two orders of magnitude. Achieving these objectives will have a major impact on our understanding of the physics of both weak and strong interactions.

## 1. Introduction

The search for an electric dipole moment (EDM) of the neutron is perhaps unique in modern physics in that experimental work on this subject has been going on more or less continuously for over 50 years. In that period the experimental sensitivity has increased by more than a factor of 10^6^ without an EDM ever being observed. The reason for this apparently obsessive behavior by a small group of dedicated physicists is that the observation of a non-zero neutron EDM would be evidence of time reversal violation. CP violation (which implies T violation by the CPT theorem) as well as T violation itself has been observed in the K^0^ and B meson systems. Both observations are however consistent with the “Standard Model” of particle physics. An interesting point is that while CP (T) violation is on the order of 10^−3^ in the K^0^ system, it is as large as 10% in the B meson system. This has led to an enormous expenditure on so-called B factories, dedicated accelerators devoted to producing high intensity B-meson beams to study CP violation with a view to check predictions of the Standard Model. However at a recent conference at Fermilab [1], the consensus among researchers in the field is that these experiments will not really be able to produce realistic tests of the Standard Model because the Standard Model, using conventional perturbation techniques, is far from being able to produce predictions of sufficient accuracy for these effects. Many of the models currently being tested by the B measurements have already been ruled out or more strongly constrained by the neutron EDM work.

An essential point is that the Standard Model predictions of the magnitude of time reversal violation are inconsistent with our ideas of the formation of the universe; namely the production of the presently observed matter/anti-matter asymmetry requires time reversal violation many orders of magnitude greater than that predicted by the Standard Model.

For these reasons Steven Weinberg has written “These electric dipole moments … seem to me to offer one of the most exciting possibilities for progress in particle physics. Experiments here … move very slowly but … there has been a lot of progress recently in calculating electric dipole moments … with results that are encouraging for future experiments.” [2]

Thus the search for a neutron EDM represents the best existing hope for finding physics beyond the Standard Model as the detection of any non-zero neutron EDM would be unambiguous proof of the breakdown of the Standard Model.

While the Noble Prize citation (1989) for the originator of experimental work on the neutron EDMs, Norman Ramsey, did not mention his neutron EDM work directly [3], it is widely felt that this work played an important role in his selection for that honor.

## 2. Present Experimental Situation

Currently there are no running neutron EDM experiments; the most recent experiment at the Institut Laue-Langevin (ILL) has now completed data collection. There are three rather large projects (including this project) engaged in design and preparation of a new generation of experiments expected to improve the experimental limit by about a factor of 100 [4,5].

Two projects, one centered at the ILL [4] and this project, a collaboration based at Los Alamos National Laboratory (LANL), involve the storage of ultracold neutrons (UCN) in superfluid Helium and cryogenic techniques for controlling the magnetic fields. The third experiment, operating at room temperature at Paul Scherrer Institut (PSI), we feel is not competitive in its present form.

The present experiment is based on the production and storage of UCN in superfluid helium doped with a very dilute solution of polarized ^3^He. The ^3^He serves as polarizer, analyzer and co-magnetometer. By this term we mean a magnetometer whose sensitive elements sense the field in the same region where the UCN are undergoing Larmor precession. If there is one essential point that has been learned during the past 25 years of experimental work in this field, it is the fact that a co-magnetometer is essential if one is to reach sensitivities comparable to or better than those currently achieved. Given this it is somewhat surprising that the LANL-based project is the only one of the current projects that offers this essential feature.

## 3. Brief Overview of the Neutron EDM

For a detailed discussion of the proposed experiment, the reader is directed to the original proposal by Golub and Lamoreaux [6] and the collaboration web page (http://p25ext.lanl.gov/edm/edm.html), where one can view the pre-proposal in its entirety. The pre-proposal contains detailed discussions of all aspects of the proposed experiment. This section serves to give a basic overview of the experiment and measurement technique.

The experiment to measure the neutron EDM is based on the magnetic resonance technique of rotating a magnetic dipole moment in a magnetic field. Polarized neutrons and polarized ^3^He atoms coexist in a bath of superfluid ^4^He at a temperature of ≈ 300 mK. When placed in an external magnetic field, both the neutron and ^3^He magnetic dipoles can be made to precess in the plane perpendicular to the magnetic field. The measurement of the neutron EDM comes from a measurement of the change in the difference in the precession frequencies of the neutrons and the ^3^He atoms when a strong electric field parallel to the magnetic field is reversed. In this comparison measurement, the neutral ^3^He atom is assumed to have a negligible electric dipole moment.

The present concept involves a superconducting cylindrical magnetic shield containing a ferromagnetic cylinder to provide a flux return path and a more spatially uniform field inside. The precession of the ^3^He nuclei will be monitored with SQUIDS and the technique of ‘dressing’ will be applied so that the magnetic moments of the ^3^He and UCN can be tuned to have effectively the same value, providing cancellation of magnetic field fluctuations and the possibility of signal to noise enhancements. In fact, the presence of two species undergoing Larmor precession makes available a wide range of double resonance techniques, well known from NMR practice.

UCNs are produced and stored in a superfluid ^4^He (He II) bath. The socalled “superthermal process” for producing UCN in superfluid ^4^He was proposed in Ref. [7] and a number of aspects of the physical processes involved have been demonstrated [8–17]. Briefly, the dispersion relation for the free neutron relating the kinetic energy *ħω* to momentum *ħk* is a parabola. This parabola crosses the Landau-Feynman dispersion curve for elementary excitations in superfluid ^4^He at 2π/k* = 0.89 nm and E* = *ħω*= 12 K. (The curves also intersect at *k* = 0.) Since both energy and momentum are conserved in the scattering process, neutrons at or near rest can only absorb phonons of energy E* where the dispersion curves cross. The rate of this process is proportional to the 12 K phonon density which is strongly suppressed by the Boltzmann factor e*^−E^* ^*/^*^kT^* and is negligibly small when the temperature of the helium bath is less than 500 mK. By the same argument, only neutrons with energy near E* can scatter into the UCN energy region through creating a single excitation.

A beam of 0.89 nm neutrons enters the superfluid bath. Some fraction of these neutrons downscatter, producing UCN which are confined by the material walls of the container if their kinetic energy is less than the effective neutron potential of the wall. If the 0.89 nm neutron beam is polarized, the UCNs produced will be polarized as well. Recent studies at the ILL have measured the polarization transfer of the incident neutron beam to UCN produced using the superthermal process and initial results indicate that the transfer is at least 99% [18].

Producing the UCN directly in the measurement region has a number of advantages. In addition to avoiding the losses associated with the transport of UCN through guides and windows, the excellent dielectric properties of liquid helium allows one to increase the applied electric field by nearly an order of magnitude as compared to the traditional experiments operated in vacuum. A dilute solution of polarized ^3^He in the ^4^He also serves as a UCN detector. When a neutron is captured by ^3^He, the energetic charged particles from this reaction produce ultraviolet scintillations in the liquid helium. The reaction between ^3^He and neutrons in the liquid helium can be detected with nearly 100% efficiency.

The spin dependent absorption rate for the neutrons by the ^3^He is given by

$$\frac{1}{\tau_{\text{abs}}}=\frac{1}{\tau_{3}}(1-p_{n}p_{3}\cos\theta_{n3})$$ (1)

where *θ*_n3_ is the angle between neutron and ^3^He spin polarization vectors, ***p***_n_ and ***p***_3_, and *τ*_3_ is the polarization lifetime of the ^3^He. Each neutron capture event produces a scintillation pulse; the scintillation rate thus becomes a measure of the angle between the polarization vectors. By measuring the precession frequency of the ^3^He using SQUID techniques, one can determine from the scintillation rate the procession frequency of the neutrons. If the neutron EDM is non-zero, the neutron precession frequency will be shifted by an amount proportional to *d*_n_*E* when the electric field *E* is reversed.

## 4. Conclusions and Discussion

In principle this new type of EDM experiment can achieve three orders of magnitude improvement in the experimental limit for the neutron EDM in conjunction with the proposed Spallation Neutron Source (SNS). This factor results from the possibility of an increased electric field (a factor of > 5) due to the excellent dielectric properties of superfluid ^4^He, an increase in total UCN stored (> 1000 fold improvement at the SNS) and an increased storage time (up to 10 times) due to the low temperature of the walls. The current experimental EDM bound, however, is largely limited by magnetic field systematics. With the proposed experiment, an EDM limit of *d*_n_ = 3 × 10^−29^ e cm is possible; the use of ^3^He as a volume co-magnetometer is crucial to the elimination of magnetic field systematics.

A recently completed work [19] and subsequent analysis [20] has called attention to the existence of a serious systematic effect produced by the combination of a magnetic field gradient and the well known ***v*** × ***E*** motional magnetic field acting on the UCN spins by the Bloch-Siegert effect. The effect is independent of the direction of the velocity. The only way so far identified to reduce the effect (other than reducing the field gradients) is through collisions with a foreign gas. The dilute solution of ^3^He acting as a co-magnetometer has the great advantage that the collision rate with the phonons in the ^4^He can be simply adjusted by small changes in the temperature and thus provides the only known method for precise in-situ monitoring of the effect.
